# Doxycycline as a Potential MMP-1 Inhibitor for the Treatment of Spondylitis Tuberculosis: A Study in Rabbit Model

**DOI:** 10.1155/2023/7421325

**Published:** 2023-01-27

**Authors:** Otman Siregar, Aznan Lelo, Ahmad Jabir Rahyussalim, Syafruddin Ilyas, Benny, Tri Kurniawati, Yohanes Augustinus, Hendra, Tommy Mandagi, Muhammad Luqman Labib Zufar, Irfan Fathurrahman

**Affiliations:** ^1^Department of Orthopaedics and Traumatology, Adam Malik General Hospital, Faculty of Medicine, Universitas Sumatera Utara, Medan, Indonesia; ^2^Department of Pharmacology and Therapeutics School of Medicine, Universitas Sumatera Utara, Indonesia; ^3^Department of Orthopaedics and Traumatology, Cipto Mangunkusumo General Hospital, Faculty of Medicine, Universitas Indonesia, Jakarta, Indonesia; ^4^Department of Biology, Faculty of Mathematics and Natural Sciences, Universitas Sumatera Utara, Medan, Indonesia; ^5^Stem Cell and Tissue Engineering Cluster, IMERI Faculty of Medicine, Universitas Indonesia, Jakarta, Indonesia; ^6^Stem Cell Medical Technology Integrated Service Unit, Cipto Mangunkusumo General Hospital, Jakarta, Indonesia; ^7^Medical graduate, Faculty of Medicine, Universitas Indonesia, Jakarta, Indonesia

## Abstract

**Background:**

Tuberculosis (TB) of the spine is a highly disruptive disease, especially in underdeveloped and developing countries. This condition requires standard TB treatment for 9–18 months, which increases patient risk of drug-resistant TB. Consequently, this raises the concern of adopting additional therapies to shorten the treatment duration, improve the efficacy of anti-TB drugs, and further decrease damage in the affected tissues and organs. Matrix metalloproteinase- (MMP-) 1 is a key regulator of the destruction of the extracellular matrix and associated proteins and is a new potential target for TB treatment research. In the present study, we investigated the effects of doxycycline as an MMP-1 inhibitor in patients with spondylitis TB.

**Methods:**

Seventy-two New Zealand white rabbits with spondylitis TB were divided into 12 different groups based on incubation period (2, 4, 6, and 8 weeks) and doxycycline administration (without, 1 mg/kg body weight (BW), and 5 mg/kg BW). We observed the course of infection through the blood concentration changes and immunohistochemical examination of MMP-1, in addition to BTA staining, culture, polymerase chain reaction (PCR), and histopathological examination.

**Results:**

Treatment with once daily 5 mg/kg BW doxycycline significantly improved the blood MMP-1 level (*p* < 0.05) compared with the placebo and 1 mg/kg BW doxycycline. A significantly reduced ongoing infection and a higher healing rate were demonstrated in rabbits with a higher doxycycline dose through BTA staining, culture, PCR, and histopathology. Various degrees of vertebral endplates, vertebral body, and intervertebral disc destruction were observed in 32 rabbits with positive histopathological findings, in addition to positive inflammatory cell infiltration, characterized by numerous lymphocytes, macrophages, and epithelial cells, as well as abundant granulation tissue and necrotic substances proximal to the inoculated vertebral area. Bone and intervertebral disc destructions were more apparent in the untreated rabbits.

**Conclusion:**

Our study demonstrated the potential of doxycycline as an adjunctive treatment in spondylitis TB. However, limitations remain regarding the differences in the pathogenesis and virulence of *Mycobacterium tuberculosis* between rabbit and human systems, sample size, and the dose-dependent effect of doxycycline. Further studies are needed to address these issues.

## 1. Introduction

The global incidence of tuberculosis (TB) is an estimated 10.4 million cases (CI 8.8 million–12 million), which is equivalent to 120 cases per 100,000 population [1]. Extrapulmonary TB (EPTB) occurs in 20% of patients with PTB. In addition, approximately 35% of EPTB cases involve osteoarticular sites, with 50% of osteoarticular TB occurring as spondylitis TB [2, 3]. Spondylitis TB is a spine infection caused by *Mycobacterium tuberculosis* that mainly results in damage to the spinal corpus and paravertebral structures in the form of bone and structural defects. This condition will further result in spinal deformity and instability disorders [4–6]. Disease transmission may be disseminated through a primary infection, in which bacteria infect the vertebra directly, or through a secondary infection via hematogenous or lymphatic spread from another infection site in the body [7, 8].

The destruction of the immensely firm structure of collagen fibrils in normal tissue is an important pathological mechanism in TB infection [9, 10]. This process is mediated mainly by proteases, among which are matrix metalloproteinases (MMP), which play various essential roles. MMPs are zinc-dependent proteases that mediate the degradation of the extracellular matrix and modulate the inflammatory response by facilitating and inhibiting various cytokines [10–12]. MMPs can degrade basement membranes and extracellular proteins. These molecules were elevated in patients with TB compared with those with other diseases or in healthy individuals. Furthermore, MMP concentration is associated with the severity of TB. This is consistent with the extent of infection as indicated by radiographic examination, cavitation formation, and the status of sputum smear examination [13–15]. Furthermore, previous studies have reported that MMP-1 is the main collagenase in TB. The upregulation of MMP-1 gene expression in primary human monocytes and macrophages has also been observed in animal models and patients with TB [9, 14, 16–19]. Faritz Siregar et al. [17] found that the MMP-1 levels in the spondylitis TB group were significantly higher than those in the spinal degenerative disease group.

Generally, medical treatment is the treatment of choice in spondylitis TB without neurological complications, with only a few special cases requiring surgical treatment. Conversely, patients with neurological complications commonly require a combination of medical and surgical treatments [2, 7]. However, the standard course of anti-TB drugs for spondylitis TB is 9–18 months. Moreover, the threat of drug-resistant TB is ever present [20, 21]. Consequently, adjunctive therapies that can reduce treatment duration, enhance the efficacy of anti-TB drugs, and control the associated damage to tissues and organs have been the subject of treatment research. Currently, doxycycline is the only approved drug with a broad range of inhibitory effects on MMPs. Doxycycline has been used in periodontal disease at sub-antimicrobial doses to suppress collagenase activity [22]. In addition, doxycycline has successfully suppressed MMP secretion by TB bacteria in monocytes and epithelial cells in the human respiratory system [15, 23, 24]. Further experiments have also shown that doxycycline decreases the replication rates of TB bacteria and enhances anti-TB drug efficacy through MMP inhibition [15, 25].

Previous studies have investigated the use of doxycycline in PTB infection, whether as monotherapy or as adjunctive treatment to standard anti-TB therapy [15, 25–28]. However, no studies have investigated the efficacy and safety of doxycycline as an MMP inhibitor in spondylitis TB. In the present study, we examined the effect of doxycycline as an MMP inhibitor in a rabbit model of spondylitis TB by measuring MMP-1 levels in serum and vertebral body tissue, as well as by examining the mycobacterial burden and extent of tissue destruction indicated by histopathological and radiographic examinations, respectively. This study investigated the potential of doxycycline as a primary or adjunctive treatment option for spondylitis TB.

## 2. Materials and Methods

### 2.1. Ethics Statement

This study investigated spondylitis TB infection in a rabbit model. The research protocol was evaluated and approved by the animal ethics committee of the Faculty of Veterinary Medicine, Bogor Agricultural University (ethic number 003/KEH/SKE/II/2020). In addition, all rabbits used in this study were provided with care and treatment according to the guidelines of the Government of the Republic of Indonesia's Regulation No. 95 of 2012 (Veterinary Public Health and Animal Welfare) and the National Guidelines on Health Research Ethics by the Health Research Ethics Committee, the Ministry of Health, Republic of Indonesia, for the welfare and treatment of laboratory animals.

### 2.2. Animals

New Zealand white rabbits were used in the study. Body weight (BW), sex, bone maturity, and the results of clinical, radiological, and laboratory examinations were used as bases for sample selection. Skeletally mature, healthy rabbits weighing 2500–3500 g were included in the investigation. Conversely, rabbits with congenital spine anomalies and/or other spine abnormalities, which may be caused by trauma, infection, or neoplasm, were excluded. Sample selection was conducted by a veterinarian with a certification in basic laboratory animal training and more than 10 years of experience in research involving laboratory animals.

### 2.3. *M. tuberculosis* Suspension

A single-cell suspension of *M. tuberculosis* (MTB) strain H37Rv was prepared in Middlebrook liquid medium. The suspension was centrifuged at 150 rpm to homogenize the mixture and incubated at 37°C for 18 hours. Sterile saline was added to dilute the suspension to obtain a bacterial cell count of 10^8^ CFU/mL, which is equivalent to 0.132 A (absorbance) of optical density measured at a wavelength of 600 nm. The preparation protocols were based on the standard operating procedures implemented in the Clinical Microbiology Laboratory of the Faculty of Medicine, Universitas Indonesia.

### 2.4. Experimental Design

**Figure 1:**
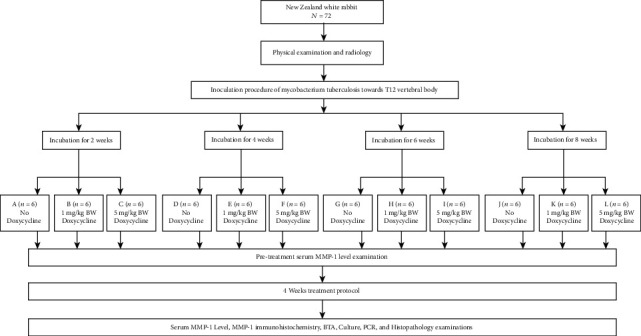
Research protocol.

Using a randomization table, MTB inoculation was randomly performed on 72 adult New Zealand white rabbits. These rabbits were divided into four major groups (*n* = 18) based on an incubation period of 2, 4, 6, or 8 weeks. These 18 rabbits were further divided into three different groups (*n* = 6), in which the first group received no doxycycline at all; the second group, doxycycline at 1 mg/kg body weight (BW); and the third group, doxycycline at 5 mg/kg BW (Figure 1). All rabbits were placed in individual cages during the observation period. All the rearing parameters and feeding regimens using pellets and rabbit feed were regulated into homogeneous conditions.

### 2.5. Preparation of the Animal Model

#### 2.5.1. Anesthesia

Anesthesia was administered via auricular vein or intramuscular injection of 3% sodium pentobarbital (30 mg/kg) mixed with ketamine HCl (44 mg/kg) and xylazine (5 mg/kg). This mixture of ketamine, pentobarbital, and xylazine was used to sufficiently stabilize the intraoperative hemodynamic parameters. The advantages of small amounts of drugs are gained without the disadvantages of large doses of any one drug. For example, ≤30 mg/kg pentobarbital does not have depressive effects on the heart compared with extremely large doses of pentobarbital that causes an antiadrenaline effect on the heart. Ketamine as cardiovascular stimulant anesthetic obtained a higher survival rate than ketamine or pentobarbital alone for anesthesia. The combination of ketamine, pentobarbital, and xylazine at the dosage used in this study reduced the concentration used for each drug to a nontoxic level [29–31].

#### 2.5.2. Inoculation of *M. tuberculosis*

The rabbits were placed in the right lateral decubitus position following the induction of anesthesia. The left side of the rabbit's back was placed facing toward the operator in charge to identify the surgical site. Hair covering the surgical site was shaved off, followed by the application of 70% alcohol and betadine solution. A sterile cloth was used as draping, leaving only the surgical site uncovered. The 12^th^ thoracic vertebra was determined by palpation toward the 12^th^ rib and its transverse processes. A 5 cm transverse skin incision was created from the center of the spinous process to the lateral area of the left back. The paraspinal muscles were separated to expose the transverse process and lamina of the 12^th^ thoracic vertebra. The 12^th^ thoracic corpus was penetrated using a 1.5 mm drill bit to make a 6 to 10 mm deep hole at the midpoint of the corpus vertebra (+5 mm from the transverses process). A 0.2 mL quantity of 10^8^ CFU/mL MTB suspension was subsequently delivered aseptically using a syringe. The hole was covered with subcutaneous fat using a root dissector. The skin incision was closed layer by layer and then covered by bandages. The rabbits were observed in their cages during postsurgical recovery. Ketoprofen was administered intramuscularly at 3 mg/kg BW every 12 hours for 3 days. The principles of standard precautions, established safety measures, aseptic technique, and animal ethics were upheld in implementing this surgical protocol [32, 33].

#### 2.5.3. Doxycycline Administration

Doxycycline was administered orally at 1 mg/kg BW and 5 mg/kg BW once daily with a 5 mL syringe to the second and third groups for doxycycline administration, respectively, with each group further subdivided based on the length of the incubation period (groups B, C, E, F, H, I, K, and L). Inoculated rabbits were given doxycycline for 4 weeks following the designated incubation periods, during which the animals were placed in individual cages in one large room. In addition, rabbits in the control groups (groups A, D, G, and J) received 3 mL saline orally [34–36].

#### 2.5.4. Study Parameters

Clinical conditions, including daily activities, signs of infection, signs of wound healing, paralysis, and appetite, were examined twice a day at 8 am and 4 pm. In addition, BW was measured every 3 days. Doxycycline was administered as an MMP-1 inhibitor for 4 weeks at the end of the 2^nd^, 4^th^, 6^th^, or 8^th^ week of incubation. A plain X-ray examination was performed for each group after the completion of doxycycline therapy. A YSRD-VET320 digital X-ray machine (China) was used at a source voltage of 56 kVp, source current of 300 mA, and exposure time of 1/30 second. The rabbits were placed in the lateral recumbent position with the fore and hind legs moderately extended cranially and caudally for the lateral spine X-ray, whereas they were placed in the dorsal recumbent position, with the forelegs extended cranially and secured with tape and the hind legs extended slightly caudally and positioned in a flexed frog position, for the ventrodorsal view. The X-ray images were graded into four categories as follows: negative, no lesion; positive 1, 1%–25% of the vertebral body; positive 2, 25%–50% of the vertebral body; and positive 3, >50% of the vertebral body. Each grade is given a particular scoring weight, as described in a previous study [32].

The rabbits were sedated using a precision vaporizer of 4.5% isoflurane in oxygen within an induction chamber and waste gas scavenger. Euthanasia was then performed with an intravenous lethal injection of 150 mg/kg BW. Samples from the infected lesions were obtained to assess the success of MTB inoculation in the vertebral bodies of the rabbits and sent on the same day for laboratory analysis. Four modalities were used for analysis, acid-fast bacilli (AFB) staining, culture, polymerase chain reaction (PCR), and histopathological examination. The samples for histopathological examination were first fixed in 10% formalin buffer. Blood samples were also obtained to check the MMP-1 level following doxycycline administration. To evaluate spinal inflammation and damage, three sections of multiple random fields were scanned for the histopathological examination. The severity was scored based on the granulomatous reaction and extent of inflammation as follows: 0 = no lesion, 1 = minimal lesion (1%–10% of vertebral body affected), 2 = mild lesion (11%–30% of vertebral body affected), 3 = moderate lesion (31%–50% of vertebral body affected), 4 = marked lesion (50%–80% of vertebral body affected), and 5 = severe lesion (>80% of vertebral body affected) [37].

A positive AFB staining was defined as 1 or more AFBs observed in 100 fields of view. A culture was positive if bacterial growth was observed in the Lowenstein–Jansen medium after 9–14 days. Positive PNB and niacin tests were required to confirm the culture findings. PCR examination was performed using MTB strain H37Rv DNA as a positive reaction control. The DNA was obtained from cultures grown on a Lowenstein–Jensen medium for 30 days. Specific primers for the MTB complex were used, including TB1-A (GAACAATCCG GAGTTGACAA) and TB1-B (AGCAGCCTGTCAATCA TGTA) oligonucleotide primers. The presence of datia Langhans cells, giant cells, caseous necrotic tissue, and other tissue reactions to MTB infection indicated a positive histopathological examination. Immunohistochemical examination was also performed to detect MMP-1. The sample was prepared in 10% neutral buffered formalin (pH 7.4) and positioned in the paraffin wax using the Histocenter. The primary antibody of MMP-1 Ab-1 was detected using a non-biotin-based kit. Brown areas indicated immunoreactivity in contrast to the blue counterstain. The samples for these examinations were obtained from the affected vertebral body, upper and lower endplates, or adjacent intervertebral disk. Nevertheless, all these assessments were performed following the International Union Against Tuberculosis and Lung Disease and World Health Organization recommendations.

#### 2.5.5. Statistical Analysis

All data collected during this study were managed using Microsoft Excel. Statistical analysis was performed using SPSS software to perform the independent *t*-test, with statistical significance set at *p* < 0.05 and 95% confidence interval.

## 3. Results

### 3.1. General Animal Conditions

All 72 rabbits survived MTB infection until the end of the incubation period and completed the experiment. All the rabbits showed stable vital signs without remarkable changes in appetite and BW. In addition, no significant adverse events, such as peritoneal rupture during surgery, postoperative paraplegia, postoperative trauma, pulmonary complications, or multiorgan failure, were observed. All the rabbits were euthanized within the scheduled time without any readjustments to the experiment protocols.

### 3.2. X-Ray Findings

**Figure 2:**
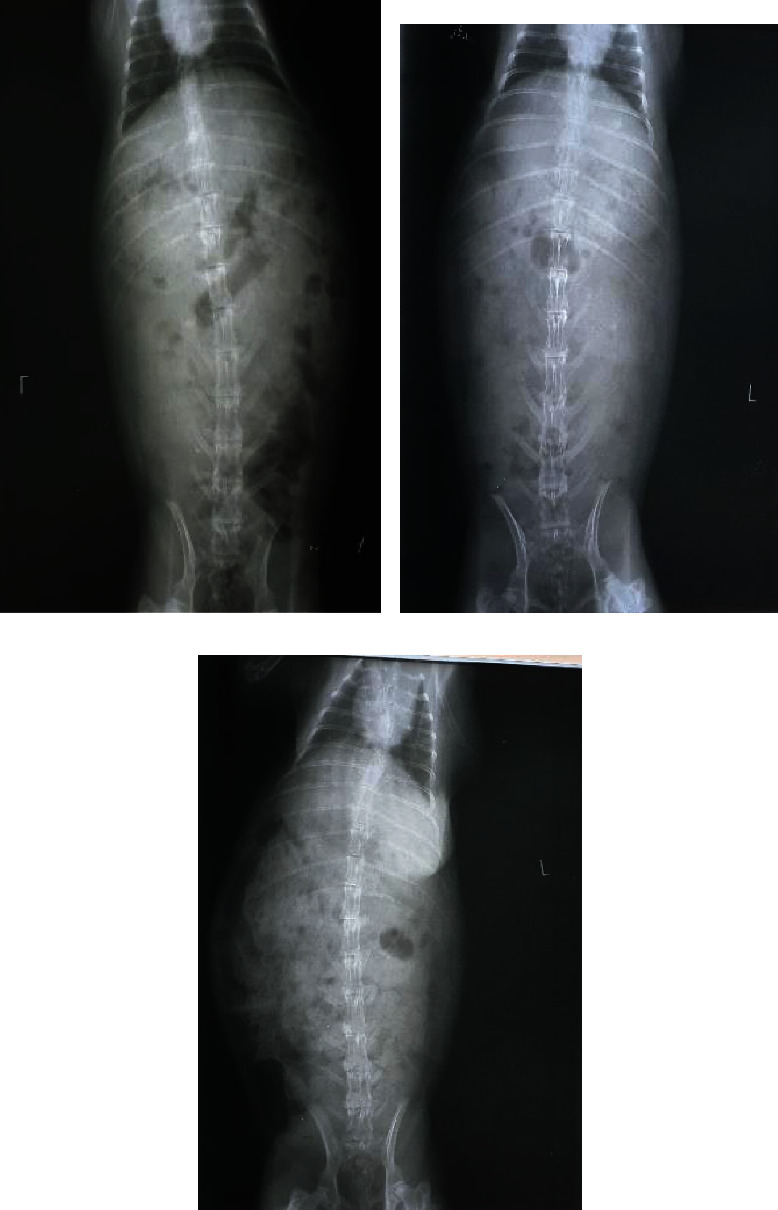
X-ray images of rabbits after 4-week incubation (a) without treatment, showing marked sclerotic changes, damaged vertebral bodies, and adjacent structures, compared with rabbits treated with doxycycline at (b) 1 mg/kg BW and (c) 5 mg/kg BW.

Slight destruction with sclerotic changes of the T12 vertebral body was observed postoperatively in the plain X-ray images starting from 4-week incubation. Radiologic examination also showed intervertebral disc changes, including slightly narrowed intervertebral spaces with slightly blurry borders with the new formation of osteophytes. The density of the corpus and endplates was relatively irregular. Marked signs of abnormal vertebral changes were also observed in the control groups, specifically groups A, D, G, and J. In contrast, rabbits treated with 5 mg/kg BW doxycycline showed less destruction across the vertebral body and adjacent structures compared with the control groups and rabbits treated with 1 mg/kg BW doxycycline (Figure 2). Comparisons of the radiological scores between groups are shown in Tables 1 and 2.

### 3.3. Gross Anatomical Observation

Varying degrees of destruction were observed across the vertebral endplates, vertebral body, and intervertebral discs in 32 rabbits with positive histopathological findings, following completion of the treatment protocols. Granulation tissue and necrotic substances were also noted proximal to the inoculated vertebra area. However, no obvious dissemination of *M. tuberculosis* was observed in the liver, lungs, or other organs on gross anatomical examination. Generally, the rabbits without doxycycline treatment showed evident bone or intervertebral destruction and paravertebral abscesses.

### 3.4. Histopathological Examination

**Figure 3:**
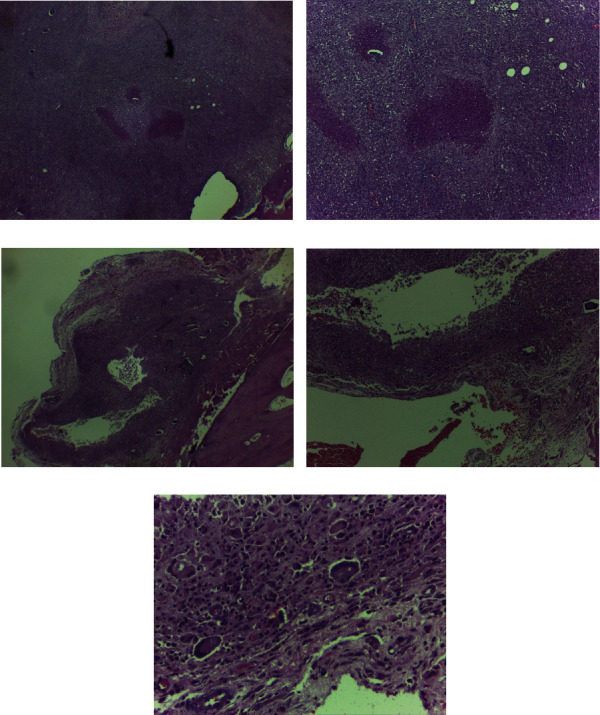
Histopathological examination with hematoxylin-eosin staining showing the presence of (a) inflammatory cell infiltration (40x magnification), (b) epithelioid cells and caseous necrosis (100x magnification), (c) tubercles (25x magnification), (d) diffused macrophage infiltration (100x magnification), and (e) datia Langhans cells (400x magnification, white arrow).

Inflammatory cell infiltration, characterized by countless lymphocytes, macrophages, limited epithelial cells, and caseous necrosis, was observed in the histopathological specimens, in addition to the bone destruction and tubercle formation in the affected vertebral region (Figure 3). Otherwise, normal trabecular bone structures with neither inflammatory cell infiltration nor epithelioid cell formation were observed in negative histopathological samples. Comparisons of the histopathological scoring of spondylitis TB infection are shown in Tables 3 and 4. Groups treated with 5 mg doxycycline obtained low histopathological scores (*p* < 0.05).

### 3.5. TB Culture

The TB cultures were positive for 32 of 72 rabbits across the 12 groups. The colonies had a slightly pale yellow appearance, as observed in the medium. No culture growth was observed in the other 40 specimens, specifically in 1 specimen from the 2-week incubation groups, 8 from the 4-week incubation groups, 15 from the 6-week incubation groups, and 16 from the 8-week incubation groups. No contamination was reported in all 72 specimens collected. In addition, the cultures for liver, lungs, spleen, and kidneys were negative, which was consistent with the findings of the gross anatomical examination.

### 3.6. MMP-1 Examination

The assessment of the MMP-1 levels was performed by measuring MMP-1 blood serum levels and by immunohistochemical examination following each incubation period and treatment protocol. The average MMP-1 blood serum levels following incubation (before treatment protocols), after the completion of doxycycline treatment, and in the positive immunohistochemical assessment for each group are shown in Table 5. Significant differences in the average MMP-1 levels were observed between groups I, H, and G, which underwent a 6-week incubation period (*p* < 0.05). The groups treated with 5 mg/kg BW doxycycline showed lower MMP-1 levels than the other groups. The immunohistochemistry results were negative for groups that underwent an 8-week incubation period (Table 5).

The rabbits in groups A, C, F, G, H, I, J, K, and L showed significant (*p* > 0.05) differences in the average MMP-1 blood serum levels before and after treatment protocols. Comparisons of blood serum MMP-1 levels between the control and treatment groups for each incubation period are shown in Tables 6 and 7.

### 3.7. Healing Rate following Doxycycline Administration

The infection and healing rates following doxycycline treatment were assessed based on the results of BTA staining, culture, PCR, and histopathological examination (Table 8). Rabbits treated with 5 mg/kg BW doxycycline showed a higher healing rate than those treated with 1 mg/kg BW doxycycline. In addition, rabbits in the control group showed a higher ongoing infection rate.

## 4. Discussion

TB remains one of the most disruptive global health problems [1–3]. *M. tuberculosis* infection of the spine induces destructive pathology that is mainly mediated by proteases, particularly, MMPs (MMP-1, -2, -3, -7, -8, -9, and -10), which is a family of zinc-dependent proteases that can collectively degrade extracellular matrix components [10–12]. Elevated MMP concentrations are commonly associated with disease severity, radiographic extent, and scarring or cavitation [13, 14, 19]. Acting as a broad-spectrum antibiotic, doxycycline is currently the most commonly available U.S. Food and Drug Administration-approved drug that suppresses MMPs [38–42]. MMPs are key regulators of tissue destruction in TB.

Doxycycline is a synthetic tetracycline that acts as an MMP inhibitor, thereby hindering connective tissue breakdown through three main mechanisms. First, mediated by extracellular mechanisms, doxycycline directly inhibits MMPs, through its Ca^++^- and Zn^++^-binding properties, and the oxidative activation of pro-MMPs, through its independent cation-binding properties. Doxycycline also protects *α*_1_-proteinase inhibitor from MMPs by indirectly lowering serine proteinase activity. Second, doxycycline participates in cellular regulation by lowering the levels of cytokines, inducible nitric oxide synthase (iNOS), phospholipase A2, and prostaglandin synthase, which are necessary to cellular breakdown. Doxycycline also reduces the levels of protein kinase C and calmodulin, which are important mediators of signal transduction that increase intracellular free Ca^++^ levels, which influence cell survival. Third, doxycycline also mediates the proanabolic effect by increasing collagen production, osteoblast activity, and bone formation [15, 38, 39].

Research on the effects of doxycycline against TB is still limited. Most studies have demonstrated the inhibitory effect of doxycycline on MMPs, but not specifically in spinal TB [15, 23, 24]. Hence, our study sought to address this research gap by examining the efficacy and safety of doxycycline as an MMP inhibitor in spondylitis TB by measuring MMP-1 levels in serum and vertebral body tissue, as well as the extent of tissue destruction via histopathological and radiographic examinations.

Our results showed significant differences in blood and IHC MMP-1 levels in our rabbit samples. MMP-1 levels increased in three groups but decreased in the other nine groups. Groups F, I, and L showed statistical significance (*p* = 0.024, 0.027, and 0.003, respectively). We also observed that the infection process, as indicated by BTA staining, culture, PCR, and histopathological examination, was significantly reduced in the treatment group with a higher doxycycline dose. Generally, the rabbits administered with the higher doxycycline dose showed better results in terms of a lower infection progress and lower MMP-1 levels. A significant difference was also observed between the groups that underwent a 2-week incubation period and those that underwent incubation > 2 weeks, both in terms of the findings of gross anatomical observation and MMP examination. Gross anatomical examination revealed greater bone destruction and abscesses in untreated rabbits.

Our results agree with those of the study conducted by Walker et al. [15] in which doxycycline suppressed MMP-1 secretion in patients with TB. Our findings are also in line with those obtained by Stechmiller et al. [41] in their investigation of doxycycline as MMP inhibitor in chronic wounds. MMP activity is controlled at the gene level by transcriptional control. At the molecular level, it is activated by the proteolytic cleavage of propertied portions or through the binding of natural inhibitors of MMPs or the tissue inhibitors of metalloproteinases (TIMPS) to active MMPs, forming stable MMP-TIMPS complexes [14, 18, 28, 41].

MMP-1 is closely related to immunopathological parameters, such as radiographic changes, cavitation, tissue infiltration by inflammatory cells, and sputum smear score, supporting the hypothesis that MMP-1 is central to TB immunopathogenesis [42]. Our findings showed higher radiological and histopathological scores in the control groups than the doxycycline-treated groups (Tables 1 and 3, *p* < 0.05) and thus support these arguments. In addition, the groups treated with 5 mg/kg BW doxycycline also showed a higher drop in radiological and histopathological scores than the control groups and groups treated with 1 mg/kg BW doxycycline.

When MTB infiltrates the corpus vertebrae in spondylitis TB, it begins breaking down the extracellular matrix. The center of TB granuloma undergoes caseous necrosis and is surrounded by activated macrophages, fibroblasts, and T cells, which later develop into an immunodeficient site where MTB can proliferate [2, 3, 9, 10]. At this site, inflammatory responses are initiated by the influx of neutrophils and macrophages and involve the action of proteases, specifically MMPs, that degrade the extracellular matrix. The vicious cycle is perpetuated by the secretion of cytokines by inflammatory cells, such as TNF-*α* and IL-1*β*, which in turn upregulate the influx of inflammatory mediators. The activation and overexpression of MMPs lead to the degradation of newly formed tissues, further delaying healing [9, 41].

MMPs are subdivided into different groups, and many have been studied. The most common grouping is based on the substrate specificity and cellular localization of MMPs, such as gelatinases, stromelysins, membrane-type MMPs, and collagenases. Collagenases can degrade triple-helical fibrillar collagens into one-fourth and three-fourth fragments that are the major components of bone and cartilages. MMPs are the only known enzymes that can degrade these components. MMP-1, along with MMP-2, -3, -9, -13, and -14, belongs in this group [9, 32–35]. MTB upregulates MMP-1, -2, -8, and -9, with MMP-1 acting as the main collagenase in TB immunopathology. The study by Rahyussalim et al. reported increased MMP-1 and -3 concentrations in patients with TB compared with patients with respiratory symptoms. MMP-1 is closely related to immunopathological parameters, such as chest radiographic infiltration, cavitation, and sputum smear score, supporting the hypothesis that MMP-1 is critical to TB immunopathogenesis [32]. In multiple studies, the upregulation of MMPs is a response to MTB infection that is driven by lipomannans and depends on Toll-like receptors and CD14 signals [9]. In our study, we found a high MMP-1 level that indicated a high ongoing infection process, as indicated by BTA, culture, PCR, and histological findings (Table 1).

The immunomodulatory strategy previously implemented to limit further progression of TB pathology was adjunctive corticosteroid treatment. Dexamethasone can decrease MMP-9 concentrations early in TB treatment, despite having little effect on other proinflammatory mediators [36, 37]. The drug is also relatively beneficial in HIV-TB immune reconstitution syndrome. However, corticosteroids have adverse effects and can be dangerous in the context of multi-drug-resistant TB. Targeting MMP activity as a common effector in immune-mediated tissue damage may thus be clinically useful in the era of multi-drug-resistant TB [9, 28, 40, 41].

Accumulating evidence has shown that doxycycline is a potential agent for suppressing MMP activity in TB that is safe, cheap, and readily available [25–28]. After a TB lesion is formed, the continued expression of MMPs and abnormal accumulation of extracellular matrix can lead to structural pathological remodeling [15–18]. Walker et al. showed that doxycycline can suppress MMP-1 and -3, along with TNF-*α* released by MTB-infected macrophages within 72 hours of initiating treatment by suppressing promoter activation. They also found that the minimum bactericidal concentration (MBC) of doxycycline was 40 *μ*g/mL with an MBC/minimum inhibitory concentration (MIC) ratio of 16, in addition to confirming the bacteriostatic character of the drug [15]. A systematic review by Alsaad et al. [27] described doxycycline as an antibiotic with a broad MMP inhibitory activity that mitigates tissue damage and suppresses disease progression in TB. These findings indicate the potential of MMP-inhibitor drugs as adjuncts to standard TB treatment regimens.

The findings of Alsaad et al. are consistent with our findings on the significant differences in the progression of infection between the treated and untreated groups. In humans, doxycycline suppresses MTB-driven MMP secretion. In our study, doxycycline lowered the blood MMP and immunohistochemistry MMP-1 levels [15, 28]. In our study, the rabbits that underwent 6- and 8-week incubation periods showed decreased MMP-1 serum levels, even without any additional doxycycline treatment. The differences in serum MMP-1 levels between these incubation period groups assessed in terms of doxycycline treatment dose were also insignificant (Table 7). This considerable reduction in blood MMP-1 levels may be due to the MTB strain used in this study, MTB H37Rv. While the strain showed promising virulence to the rabbit, it lacks longevity, with the peak of virulence occurring at around weeks 5–7. These characteristics may result in lesser inflammation after 7 weeks, which would explain the decrease in MMP-1 blood levels during the 8th and 12th weeks (Tables 5–7) [36, 43]. These claims are also supported by the higher radiological and histopathological scores for the groups with longer incubation periods, except those that underwent 8-week incubation, as shown in Tables 1–4.

In our study, we also found that doxycycline suppressed the positivity rate in the histopathological examination. The reduction in positive numbers was mainly in group L (0% positive rate on histopathological examination; underwent an incubation period of 8 weeks and received doxycycline 5 mg/kg BW/day for 4 weeks). These findings are consistent with those of Walker et al. [15], who found that doxycycline could reduce MMP-induced tissue destruction in TB. In addition, the results also showed that doxycycline can suppress the number of colony-forming units of TB in the lungs, which is positively correlated with the percentage of guinea pig lung granulomatous infiltrates.

The study by Lucateli et al. showed that the oral administration of penetration in rabbits has the poorest absorption compared with administration by intravenous bolus or subcutaneous injection. However, moderate uptake in macrophages is still favorable in reaching a good MTB MIC. Doxycycline was reported to form calcium-bound depots that are localized in bones and teeth which contain 99% of the body's calcium [44]. This effect of doxycycline enhanced healing in MTB-infected rabbit spine (Table 2) where all groups that received 5 mg/kg BW doxycycline (groups C, F, I, and L) yield negative immunohistochemistry tests. Doxycycline also has a positive effect on bone regeneration, as its inhibition of collagenolytic enzymes is amplified by MMP-1 in the MTB infection setting. The study by Webster and del Rosso [45] showed that doxycycline induced a significantly high bone formation rate in rats with critical size bone defects. Doxycycline-treated rats also showed 10% new bone formation at the margin of the defects. In our study, fewer necrotic substances were found in the rabbit groups treated with doxycycline, whereas more apparent bone destruction and abscesses were observed in the untreated ones.

A previous study also showed that tetracycline offers pharmacologic intervention beyond its antimicrobial profile, thereby exhibiting the potential to neutralize these unwanted matrix components [46, 47]. Furthermore, other more specific MMP inhibitors than doxycycline have been developed, such as Ro32-3555, a potent collagenase inhibitor commonly used in noninfectious diseases that has been proven safe for human treatment [48, 49]. MMP inhibitors, including doxycycline, should be evaluated further as adjunctive agents in TB treatment that can reduce immunopathology [9].

Our literature search did not retrieve any similar studies that examined the effect of doxycycline in suppressing MMPs in patients with spondylitis. However, several papers have studied the suppressive effects of doxycycline on MMPs. A study by Stechmiller et al. [41] showed that doxycycline is highly effective in treating chronic wounds, given its potent ability to inhibit MMPs. Similarly, Altoé et al. [50] examined tissue repair in rats with doxycycline use and found that MMP levels in doxycycline-treated rats with subcutaneous exudate pouches after croton oil injection were lower compared with the untreated group. Xu et al. [25] also showed that doxycycline modulates antioxidant defense, thereby accelerating wound healing in rats.

MTB is exclusively pathogenic to humans; thus, most animal models would not be able to replicate all pathological features that occur with MTB infection in humans. A study by Elkington et al. showed that MMP expression was upregulated in human lungs, especially MMP-1 and MMP-9 [9, 18]. However, in nonhuman subjects (primates and rabbits), MMPs were upregulated 4–6 weeks after infection [50]. Xu et al. found that the broad-spectrum MMP-inhibitor marimastat enhanced the efficacy of frontline TB drugs, specifically rifampicin and isoniazid, by exhibiting synergistic activities. They further compared the MMP levels between caseous human pulmonary TB granulomas and normal lung parenchyma. The enhanced bactericidal properties resulted in increased drug delivery and improved blood vessel health [25].

Our study has several limitations. First, this study was limited by the number of rabbits used during the study. We also lacked a proper control group that received doxycycline treatment at the U.S. FDA-recommended dose of 3 mg/kg BW, which could have provided an added comparison for the treatment doses implemented in this study. Despite knowing the bioavailability of doxycycline was highest when administered intravenously, we did not examine this as a control. Furthermore, although we randomized and divided the animals, we did not set a base measurement for MMP-1 level. This resulted in a broad range of blood MMP-1 levels obtained that may have influenced pre- and posttreatment blood MMP-1 levels, although we tried to quantify the data by IHC. Furthermore, the MTB strain used has a peak inflammatory characteristic at around 4–6 weeks of infection, hence influencing the MMP levels measured for the groups that underwent an 8-week incubation period. Nevertheless, our study is the first to investigate the effect of doxycycline in a rabbit model of spondylitis TB. Our findings can serve as baseline information for future research on doxycycline as adjunctive therapy for TB treatment, especially in human spondylitis TB studies.

## 5. Conclusion

This showed that doxycycline administered at 1 and 5 mg/kg BW/day for 4 weeks both significantly decreased serum MMP-1 levels, especially in rabbits that had been inoculated for 6 and 8 weeks. Doxycycline can mitigate the destruction of vertebral body tissue by tuberculous spondylitis by interrupting infection progression, as indicated by the findings of BTA, culture, PCR, and histological testing. Doxycycline is widely available, safe, and cheap and is currently the only approved MMP inhibitor on the market. It shows potential as an adjunctive treatment in spondylitis TB. However, this study is limited by the number of samples collected, differences in the pathogenesis and virulence of MTBs between rabbits and humans, and the dose-dependent action of doxycycline as an antibacterial and anti-inflammatory drug against MTB. Further studies are needed to sufficiently address these issues.

## Figures and Tables

**Table 1:** Radiological scoring of spondylitis TB infection for each treatment group.

Group	Mean ± SD	*p* value
A	9.00 ± 2.45	<0.001^a^
B	0	<0.001^b^
C	0	NA^c^
D	11.00 ± 3.29	0.032^a^
E	6.67 ± 3.27	0.026^b^
F	4.00 ± 4.38	0.044^c^
G	13.00 ± 4.52	0.039^a^
H	8.00 ± 0.00	0.012^b^
I	5.33 ± 4.13	0.042^c^
J	6.67 ± 3.27	<0.001^a^
K	0	<0.001^b^
L	0	NA^c^

^a^ *p* value between the control group and the group treated with 1 mg/kg body weight doxycycline once daily. ^b^*p* value between the control group and the group treated with 5 mg/kg body weight doxycycline once daily. ^c^*p* value between the groups treated with 1 and 5 mg/kg body weight doxycycline once daily. SD: standard deviation; NA: not applicable. Given the score = 0 for groups B, C, K, and L.

**Table 2:** Comparison of radiological scoring between control groups for each incubation period (*p* value).

Group	A	D	G	J
A	NA	0.048	0.023	0.038
D	0.048	NA	0.047	0.031
G	0.023	0.047	NA	0.034
J	0.038	0.031	0.034	NA

NA: not applicable.

**Table 3:** Histopathological scoring of spondylitis TB infection for each treatment group.

Group	Mean ± SD	*p* value
A	2.50 ± 0.55	0.029^a^
B	1.00 ± 0.63	0.018^b^
C	0.67 ± 0.52	0.121^c^
D	3.00 ± 0.89	0.049^a^
E	2.00 ± 0.89	0.031^b^
F	1.17 ± 0.98	0.048^c^
G	1.33 ± 1.51	0.013^a^
H	0.33 ± 0.82	0.012^b^
I	0.17 ± 0.41	0.005^c^
J	0.17 ± 0.41	NA^a^
K	0.17 ± 0.41	NA^b^
L	0.17 ± 0.41	NA^c^

^a^ *p* value between the control group and the group treated with 1 mg/kg body weight doxycycline once daily. ^b^*p* value between the control group and the group treated with 5 mg/kg body weight doxycycline once daily. ^c^*p* value between the groups treated with 1 and 5 mg/kg body weight doxycycline once daily. SD: standard deviation; NA: not applicable.

**Table 4:** Comparison of histopathological scoring between the control groups for each incubation period (*p* value).

Group	A	D	G	J
A	NA	0.121	0.042	0.025
D	0.121	NA	0.038	0.013
G	0.042	0.038	NA	0.032
J	0.025	0.013	0.032	NA

NA: not applicable.

**Table 5:** MMP-1 serum levels and immunohistochemical examinations for spondylitis TB.

Group	Blood levels of MMP-1			Immunohistochemistry of MMP-1	
	Before	After	*p* value	Positive	Negative
A	0.309	0.531	0.048	4	2
B	0.298	0.373	0.251	3	3
C	0.287	0.193	0.062	2	4
D	0.398	0.439	0.352	2	4
E	0.389	0.211	0.116	1	5
F	0.406	0.117	0.024	1	5
G	0.521	0.269	0.079	1	5
H	0.511	0.139	0.037	1	5
I	0.526	0.121	0.027	1	5
J	0.462	0.131	0.037	0	6
K	0.421	0.116	0.041	0	6
L	0.457	0.091	0.033	0	6

^∗^ *p* value between before and after treatment with doxycycline for 4 weeks.

**Table 6:** Comparison of blood level of serum MMP-1 between control groups for each incubation period (*p* value).

Group	A	D	G	J
A	NA	0.121	0.038	0.087
D	0.121	NA	0.049	0.119
G	0.038	0.049	NA	0.254
J	0.087	0.119	0.254	NA

NA: not applicable.

**Table 7:** Comparison of blood serum MMP-1 levels between groups treated with 1 and 5 mg/kg body weight doxycycline.

Group	Incubation period	*p* value
B and C	2 weeks	0.021
E and F	4 weeks	0.037
H and I	6 weeks	0.348
K and L	8 weeks	0.362

**Table 8:** Infection process following treatment protocols in rabbits with spondylitis TB.

Group	Results after treatment protocols				Infection process	
	BTA	Culture	PCR	Histology	Ongoing	Resolved
A	4	6	6	6	91.67	8.33
B	4	5	5	5	79.17	20.83
C	3	4	4	3	58.33	41.67
D	4	4	6	5	79.17	20.83
E	3	4	4	4	62.50	37.50
F	1	2	2	1	25.00	75.00
G	1	2	3	3	37.50	62.50
H	1	1	2	1	20.83	79.17
I	0	0	1	1	8.33	91.67
J	0	1	1	1	12.50	87.50
K	0	0	1	1	8.33	91.67
L	0	0	1	1	8.33	91.67

PCR: polymerase chain reaction.

## Data Availability

The data used to support the findings of this study are available from the corresponding author upon request.
